# New update to the guidelines on testing predictive biomarkers in non-small-cell lung cancer: a National Consensus of the Spanish Society of Pathology and the Spanish Society of Medical Oncology

**DOI:** 10.1007/s12094-022-03046-9

**Published:** 2022-12-26

**Authors:** Dolores Isla, Maria D. Lozano, Luis Paz-Ares, Clara Salas, Javier de Castro, Esther Conde, Enriqueta Felip, Javier Gómez-Román, Pilar Garrido, Ana Belén Enguita

**Affiliations:** 1 Lozano Blesa University Clinical Hospital, IIS Aragón, Spanish Society of Medical Oncology (SEOM), C. de San Juan Bosco, 15, 50009 Zaragoza, Spain; 2 Clínica Universidad de Navarra, Spanish Society of Cytology (SEC), Spanish Society of Pathology (SEAP), Pamplona, Spain; 3 12 de Octubre University Hospital, Spanish Society of Medical Oncology (SEOM), Madrid, Spain; 4 Puerta de Hierro University Hospital, Spanish Society of Pathology (SEAP), Madrid, Spain; 5 La Paz University Hospital, Hospital La Paz Institute for Health Research (IdiPAZ), Spanish Society of Medical Oncology (SEOM), Madrid, Spain; 6 12 de Octubre University Hospital, Research Institute 12 de Octubre University Hospital (I+12), Spanish Society of Pathology (SEAP), Madrid, Spain; 7 Vall d’Hebron University Hospital, Spanish Society of Medical Oncology (SEOM), Barcelona, Spain; 8 Cantabria University, Marqués de Valdecilla University Hospital, Valdecilla Biomedical Research Institute (IDIVAL), Spanish Society of Pathology (SEAP), Santander, Spain; 9 Ramón y Cajal University Hospital, Spanish Society of Medical Oncology (SEOM), Madrid, Spain; 10 12 de Octubre University Hospital, Spanish Society of Pathology (SEAP), Madrid, Spain

**Keywords:** *ALK*, Biomarkers, *BRAF*, *EGFR*, Non-small cell lung cancer, PD-L1, *ROS1*

## Abstract

Non-small cell lung cancer (NSCLC) presents the greatest number of identified therapeutic targets, some of which have therapeutic utility. Currently, detecting *EGFR*, *BRAF*, *KRAS* and *MET* mutations, *ALK*, *ROS1*, *NTRK* and *RET* translocations, and PD-L1 expression in these patients is considered essential. The use of next-generation sequencing facilitates precise molecular diagnosis and allows the detection of other emerging mutations, such as the *HER2* mutation and predictive biomarkers for immunotherapy responses. In this consensus, a group of experts in the diagnosis and treatment of NSCLC selected by the Spanish Society of Pathology and the Spanish Society of Medical Oncology have evaluated currently available information and propose a series of recommendations to optimize the detection and use of biomarkers in daily clinical practice.

## Introduction

Non-small cell lung cancer (NSCLC) is a group of tumours with the greatest number of identified therapeutic targets, some of which have clinical utility from the earliest stages. Undoubtedly, providing the correct molecular diagnosis is required to offer the best therapeutic option to each patient, which should be applied as widely as possible. Fortunately, in recent years, important advances in both molecular diagnostic techniques and personalized therapies have been achieved. This document aims to offer new recommendations for the detection of predictive biomarkers of NSCLC and will be an update to those already published in 2012, 2015 and 2020 as a result of this consensus between the Spanish Society of Medical Oncology (SEOM) and the Spanish Society of Pathology (SEAP) [1].

## Requirements for the analysis of an optimal biological sample

Several types of samples can be useful for the study of biomarkers, such as biopsies, surgical specimens and/or cytology, as long as they contain a sufficient number of tumour cells and have been correctly processed [2, 3]. The decision about which one to consider will depend on the experience and technologies available at each laboratory. In general, using the most recent sample is recommended, especially in previously treated patients [4].

The sample should be stored in 10% buffered neutral formalin for 6–48 h depending on its size (6–12 h for small samples and 24–48 h for surgical resections), and a minimum of 50–100 cells that are viable for immunohistochemistry (IHC) and fluorescence in situ hybridization (FISH) studies should be present. The use of alternative fixatives, such as mercury-based or alcohol-based fixatives, should be avoided. For cytological samples, the cell block is processed exactly like a biopsy. Smears are fixed in 96% alcohol and should be stained with Papanicolaou. From these materials, most biomarker studies can also be performed [5]. For techniques based on nucleic acid extraction, the threshold of analytical sensitivity, the limit of detection (LOD), of the method used must be known. Each type of technique has different minimum requirements, including 30% tumour cells for direct sequencing, 5% for real-time polymerase chain reaction (PCR) and 20% for next-generation sequencing (NGS) [6]. In addition, the type of mutation can change the sensitivity threshold. Thus, a range of nucleic acid content between 5 and 10% is required to detect point mutations and small insertions or deletions, and a range up to 30% is needed to correctly analyse alterations in the number of copies [6]. Two alternative methods for redundant molecular detection are recommended, if necessary.

Regarding the management of all types of biological samples, protocols that allow both anatomopathological diagnosis and biomarker detection are required.

## What biomarkers should be tested for in NSCLC?

Table 1 shows the biomarkers that must be detected in patients with NSCLC, and Table 2 provides other biomarkers of interest in these patients.

**Table 1:** Essential biomarkers in NSCLC patients

Gene/protein	Predictive alteration	Methodology
*EGFR*	Mutation	PCR: Sanger sequencing, real-time PCR and NGS
*ALK*	Rearrangement	IHC, FISH, real-time PCR and NGS
*ROS1*	Rearrangement	IHC (screening), FISH, real-time PCR and NGS
*BRAF* V600	Mutation	Real-time PCR and NGS
PD-L1	Overexpression	IHC
*NTRK*	Rearrangement	IHC (screening), real-time PCR and NGS
*RET*	Rearrangement	FISH, real-time PCR and NGS
*KRAS*	Mutation	PCR: Sanger sequencing, real-time PCR and NGS
*MET*	Mutation Amplification	NGS FISH, real-time PCR and NGS

*ALK* anaplastic lymphoma kinase, *BRAF* B-Raf proto-oncogene, *EGFR* epidermal growth factor receptor, *FISH* fluorescence in situ hybridisation, *IHC* immunohistochemistry, *KRAS* kirsten rat sarcoma virus, *MET* mesenchymal epithelial transition factor, *NGS* next-generation sequencing, *NSCLC* non-small cell lung cancer, *NTRK* neurotrophic tyrosine receptor kinase, *PCR* polymerase chain reaction, *PD-L1* programmed death ligand-1, *RET* rearranged during transfection, *ROS1* c-ros oncogene 1

**Table 2:** Other biomarkers of interest in NSCLC patients

Gene/protein	Predictive alteration	Methodology
*HER2*	Mutation Amplification	NGS FISH, real-time PCR, NGS
TMB	Mutations	NGS
*STK11*	Mutation	NGS
*KEAP1*	Mutation	NGS
MSI	Pattern of hypermutation	IHC, PCR, NGS

*FISH* fluorescence in situ hybridisation, *HER2* human epidermal growth factor receptor 2, *IHC* immunohistochemistry, *KEAP1* Kelch-like ECH-associated protein 1, *MSI* microsatellite instability-high, *NGS* next-generation sequencing, *NSCLC* non-small cell lung cancer, *PCR* polymerase chain reaction, *STK11* serine/threonine kinase 11, *TMB* tumour mutation burden

### EGFR

Mutations in the epidermal growth factor receptor (*EGFR*) gene are identified in approximately 10–16% of NSCLCs, with a higher frequency among patients with adenocarcinoma and nonsmoking patients [7]. The most frequent mutations that are directly related to sensitivity to anti-*EGFR* tyrosine kinase inhibitors (TKIs) affect exon 19 and consist of deletions that preserve the reading frame (in-frame deletions) between codons 746 and 759 (amino acids leucine, arginine, glutamate and alanine, LREA) (45–50%), followed by missense point mutations in exon 21, with substitution of the amino acid leucine with arginine at position 858 (L858R) (35–45%). Several EGFR-TKIs have been approved for the first-line treatment of patients with metastatic disease and *EGFR*-activating mutations (exon 19 deletion, L858R) [8], including osimertinib (the preferred option in most guidelines), gefitinib, erlotinib, afatinib and dacomitinib. Osimertinib is also approved as an adjuvant treatment after complete surgical resection in adult patients with *EGFR*-activating mutations [9].

Other mutations, such as insertions in exon 20, should also be detected due to their different effects in the disease, and treatment requirements [9].

In relation to the methodology, the clinical tests for *EGFR* detection should be able to detect all the individual mutations that have been reported with a frequency of at least 1% in *EGFR*-mutated NSCLC. Highly sensitive methods are recommended. Regarding the result reports, the mutations that have been detected and the sensitivity of the detection methods used should be specified, among other data [3].

The initial recommendations for the diagnosis of mutations in *EGFR* have undergone some changes, including the fact that any cytological sample with adequate cellularity and preservation can be used, the need to use techniques with high sensitivity compared to Sanger sequencing as the reference method and the lack of sensitivity of IHC for the diagnosis of mutations in clinical practice [7].

Most patients with sensitizing *EGFR* mutations (exon 19 deletion and exon 21 mutations, L858R) receive an anti-*EGFR* TKI regimen, with the most frequent molecular mechanism of acquired resistance being the *EGFR* T790M mutation in patients receiving first- or second-generation anti-*EGFR* TKIs (50–60% of cases). Techniques that can detect this mutation in at least 5% of viable cells should be used, including new digital PCR methods [10].

The mechanisms leading to acquired resistance against TKIs vary, including intragenic mutations, gene amplification or fusion and functional adaptation with histological transformation. Accordingly, mechanisms of acquired resistance should be monitored using tumour biopsy or liquid biopsy (LB) [11].

### ALK

Anaplastic lymphoma kinase (*ALK*) rearrangements are present in 2–5% of advanced NSCLCs [9]. These tumours are arising more often in younger patients, and females with or without minimal prior tobacco smoking exposure. The disease is frequently aggressive in its clinical course and presents with thromboembolic events, and metastases in the liver, serosal surfaces and brain are common [12]. Outcomes, including survival, are dramatically improved with specific ALK TKIs and, at present, median overall survival for stage IV patients frequently exceeds 5 years. Crizotinib was the first drug approved in this context and, since then, second- (ceritinib, alectinib and brigatinib) and third-generation (lorlatinib) TKIs are available in the European Union for the treatment of untreated patients and for those following progression on prior inhibitors [13]. The benefit of individual drugs in these pretreated patients depends on the mechanism of resistance, which frequently involves acquired mutations in the ALK kinase [14].

The histological types eligible for *ALK* rearrangement tests should include all adenocarcinomas, carcinomas with non-squamous histological evidence and squamous tumours in patients younger than 50 years of age and/or with low or no tobacco exposure (i.e. < 10 pack-years) [15]. The key methods for detecting *ALK* gene rearrangement are IHC, FISH and NGS. At present IHC represents a fast, reliable and cost-effective method to detect ALK fusions [16]. Its use in cytology smears is quite controversial, although recent studies have proven the suitability of the method [5]. The most commonly used antibodies for detecting rearrangements are D5F3 (Ventana^®^ *ALK* [D5F3] CDx Assay, Tucson, Arizona, USA) and 5A4 (Novocastra^®^, Leica Biosystems^®^, Buffalo Grove, Illinois, USA), although the latter is not included in a diagnostic kit [17]. The cecal appendix is suitable as both a positive and a negative control. It must be fixed and processed under the same conditions as the patient sample. A positive tumour case can also be used as a control.

The role of FISH as the optimal standard methodology is currently controversial, although there are automated reader algorithms approved by the United States Food and Drug Administration (FDA) that greatly increase reliability [18]. When there is a positive IHC result as manifested by strong granular cytoplasmic staining with either of the 5A4 or D5F3 antibodies, confirmation by a second technique is not mandatory [15]. However, it is strongly advisable in cases that are inconclusive. This diagnostic redundancy is also helpful if unusual FISH staining is found [19].

Lastly, the methods based on NGS and RNA assays are highly specific and there are numerous studies that demonstrate their value for detecting fusions in patients who show negative results with other techniques [19]. Variant testing for specific rearrangements in *ALK*, which may provide some useful information in terms of predicting response to specific inhibitors, does not yet have sufficient data for recommendation, although it could be useful in the future [9, 14, 19]. In some circumstances, LB may replace tissue tumour biomarker analysis, and *ALK* profiling in circulating tumour DNA (ctDNA) may serve as a treatment guiding tool [20, 21].

*ALK* mutations are emerging as important resistance mechanisms to *ALK* TKIs, and *ALK* mutation testing in this scenario may provide crucial treatment guiding information as newer-generation *ALK* TKIs display different efficacies against different *ALK* mutations [22].

### ROS1

The c-ros oncogene 1 (*ROS1*) encodes a receptor with tyrosine kinase activity. Activating gene rearrangements with several partner genes are found in approximately 1% of NSCLCs, particularly those arising in young, nonsmoking patients [23]. These tumours are frequently associated with thrombotic events and have the propensity to develop central nervous system (CNS) metastases [24]. *ROS1* fusions occur almost exclusively in adenocarcinomas, frequently in those with a solid component and signet-ring cells [25]. This histological profile is also typical of tumours harbouring an *ALK* translocation. Indeed, both receptors have a 77% similarity in their ATP-binding domain.

Crizotinib was the initial TKI approved for the first- or second-line treatment of stage IV lung cancer patients with *ROS1* rearrangement [26]. More recently, TKIs such as lorlatinib, entrectinib and repotrectinib are being studied but are not yet approved for this indication [27].

Currently, it is recommended to carry out *ROS1* testing in patients with advanced stage lung adenocarcinoma, regardless of the clinical characteristics. *ROS1* testing is not recommended in squamous cell carcinoma, except in low or light smokers [27]. Three technologies are used to detect the following *ROS1* rearrangements: IHC, cytogenetic techniques, particularly FISH [9], and molecular techniques such as reverse transcription PCR (RT-PCR) and particularly NGS [15, 19]. IHC is generally recommended as a screening method and positive cases should be confirmed with another orthogonal method (e.g., FISH or NGS), due to the variable specificity of the two commercially available antibodies (D4D6, Cell Signalling Technology and SP384, Ventana Medical Systems^®^) [3, 15, 28]. The specimen for analysis should include at least 20 tumour cells and each laboratory should validate its own interpretation range [15, 19, 28]. An external control must be available, other than cecal appendix, and it is advisable also to have a positive tumour control. The existence of positive peritumoural reactive pneumocytes has also been considered as a control. Of note, *ROS1* expression, typically focal, can be found in up to one-third of tumours without underlying *ROS1* rearrangements, but with other genomic alterations (e.g., mutations of *EGFR*, kirsten rat sarcoma virus [*KRAS*], *BRAF* or human epidermal growth factor receptor 2 [*HER2*], and *ALK* rearrangements) [28, 29]. In addition, non-specific immunostaining has also been observed in the histological subtype of infiltrating mucinous adenocarcinoma and in non-tumour tissue [30].

FISH is one of the reference techniques. It uses dual-colour break-apart probes and a count of at least 50 tumour cells is recommended [15, 28, 30, 31]. A tumour should be considered positive when at least 50% of tumour cells have break-apart signals (separated by ≥ 1 signal diameter), and/or 3’ isolated signals (frequently marked with green fluorochrome) [30]. False positives and false negatives have been described, attributable to both methodological and biological causes [30, 32]. Last, NGS technologies (DNA or RNA-based), have shown high sensitivity and specificity in tumour samples and also in ctDNA [20, 21].

### BRAF

Mutations of the B-Raf proto-oncogene (*BRAF*) are observed in 2% of lung carcinomas, are exclusive to other tumour types and appear mostly in adenocarcinomas, especially of the papillary type (80%) [33]. The most frequent mutation is *BRAF*^*V600E*^ (Val600Glu) (50%), which predominates in women and may entail greater tumour aggressiveness, while the rest are more common in men or patients who smoke [34]. The European Medicines Agency (EMA) and the FDA approved dabrafenib and trametinib after their efficacy was demonstrated in phase II clinical trials in patients with the *BRAF*^V600^ mutation [9]. In the case of the FDA, the approval includes the need to detect the mutation with the NGS Oncomine Dx Target Test^®^ panel [35].

Currently, any PCR method with adequate sensitivity and quality to identify *BRAF* mutations is allowed. However, detection of this mutation individually is not recommended, so this mutation is usually studied in NGS panels, which analyse at least exons 11 and 15 of that gene.

### PD-L1

Immune checkpoint inhibitors (ICIs), programmed cell death protein-1/ligand-1 (PD-1/PD-L1) inhibitors and to a lesser extent cytotoxic T-lymphocyte-associated protein 4 (CTLA-4) blockers, have proven to be an effective strategy in the treatment of lung cancer, NSCLC and small cell lung cancer (SCLC), over the past 15 years [36]. At present, randomized clinical trial data support them as the standard treatment for patients with locally advanced or metastatic NSCLC, whether in monotherapy or in combination with chemotherapy [37]. More recently, PD-1/PD-L1 blockade has been shown to be also effective in the adjuvant and neo-adjuvant context in patients with early disease [38, 39]. Although PD-L1 is far from being an ideal biomarker, the magnitude of benefit from PD-1/PD-L1 blockers in monotherapy is related to the tumour expression of PD-L1 [38]. In contrast, PD-L1 expression does not predict the efficacy of combination regimens of chemotherapy plus PD-1/PD-L1 inhibitors, PD-1 plus CTLA-4 blockers or chemotherapy plus PD-1 plus CTLA-4 blockers [37].

PD-L1 testing is based on IHC, currently the only validated predictive test. The diversity of IHC assays and cut-off points that define a positive result have been a source of confusion and have driven a number of harmonizing efforts by the scientific community [40, 41]. Current guidelines for the determination of the PD-L1 biomarker recommend the usual pre-analytical conditions of IHC testing. PD-L1 expression is evaluated by determining the percentage of tumour cells with partial or full membrane staining of any intensity.

There are several PD-L1 clones available for IHC testing. The four most widely used in pathology laboratories are 22C3 and 28–8 by Agilent (which share the Autostainer LINK 48^®^ diagnostic platform by Agilent^®^), SP263 by MedImmune^®^/Ventana^®^, and SP142 by Spring^®^/Bioscience^®^/Ventana^®^ (which share the Ventana^®^ BenchMark diagnostic platform) [1]. The performance characteristics of the 22C3 and 28–8 assays appear to be similar based on side-by-side evaluation in retrospective cohorts. SP263 and E1L3N, used in routine practice but not approved as companion diagnostic tests, can show comparable patterns of staining to the approved assays when properly validated. The one consistent outlier has been the SP142 assay, which shows lower tumour cell staining, despite the fact that SP142 antibody recognizes identical or nearly identical epitopes as SP263 and E1L3N [42]. The SP142 assay was reportedly optimized for both tumour cell and immune cell scoring. However, its performance as an immune cell marker is further confounded by poor interobserver agreement in the interpretation of immune cell expression [43].

Regarding sample selection, if more than one tissue block is available for a given tumour, the most representative sample should be tested. More than one block may be tested when the reporting pathologist determines that additional testing is necessary to establish the tumour’s PD-L1 status. If additional blocks from the same sample are tested, the results from all tested blocks should be combined as though testing had been carried out in a single paraffin block [44].

It is not uncommon for the only material available to come from cytology samples. In these cases, it should be noted that the use of PD-L1 IHC kits that are validated for formalin-fixed paraffin-embedded (FFPE) biopsy samples, and not specifically for cytology samples, may be used if the cytology samples were processed according to the same pre-analytical conditions as required by the kits [45].

### NTRK

Fusions of neurotrophic tyrosine receptor kinase (*NTRK*) can be present in a wide variety of tumours, in both adults and paediatric patients, with the estimated frequency in NSCLC being less than 1%. Most are found in adenocarcinomas, and the most frequently involved gene is *NTRK1* [9].

No clinical or pathological data characterise affected patients, but identifying them is crucial since tropomyosin receptor kinase inhibitors (iTRKs) are available, such as larotrectinib and entrectinib, which have been approved by the EMA and the FDA for the treatment of tumours with *NTRK* fusions [46]. Despite the marked efficacy, resistance often develops, and clinical results for second-generation iTRKs are already available [47].

Two following strategies are recommended to detect these alterations: NGS with a panel including the study of the three genes (i.e., *NTRK1, 2* and *3*), and an adequate number of rearrangement pairs, or screening by IHC with mandatory subsequent confirmation of all positive results obtained by NGS [48, 49].

### RET

Fusions of the gene rearranged during transfection (*RET*) are observed in different types of tumours, with a frequency of 1–2% in NSCLC, mainly in adenocarcinomas in nonsmoking patients. *KIF5B* is the most frequent rearrangement pair [50]. The presence of calcifications in the form of psammoma bodies should suggest *RET* fusions [51].

Currently, selective inhibitors are available, such as selpercatinib and pralsetinib, which have high response rates, although their approval by regulatory agencies for first-line treatment in patients with advanced disease is conditional on the results obtained in ongoing phase III studies [52, 53].

The optimal detection method for *RET* fusion is NGS, but FISH or PCR can also be used [54]. Regarding the design of an efficient fusion search algorithm in *RET*, it is important to consider the following: (1) NGS based on the study of RNA is more sensitive than if only DNA is studied, and (2) the results of FISH can be difficult to interpret [55, 56].

### KRAS

Mutations in *KRAS* are identified in 25% of patients with NSCLC. They are found in all histological subtypes of adenocarcinoma, although they are more common in the invasive mucinous variant. They are also detected in 5% of squamous tumours [57]. Their presence confers biological and clinical heterogeneity and may have no prognostic value [58].

Mutations in *KRAS* are usually located in codons 12, 13 and 61. Mutations in codon 12 account for 80% of cases and are generally substitutions of glycine by cysteine (*KRAS*^G12C^), valine (*KRAS*^G12V^) or aspartic acid (*KRAS*^G12D^), with frequencies of 10–13%, 5 and 4%, respectively [59]. *KRAS*^G12C^ and *KRAS*^G12V^ mutations are usually related to smoking and activate the RalGDS/Ral/FLIP pathway, while the *KRAS*^G12D^ mutation is more typical in nonsmoking patients and seems to activate the PI3K/AKT/mTOR and RAF/MEK/ERK pathways. In addition, *KRAS*^G12C^ shows greater phosphorylation of ERK1/2.

More than 50% of NSCLCs with *KRAS* mutations present another mutation, and three subgroups can be established: the KP subgroup has mutations in tumour protein 53 (TP53) and represents 40% of cases; the KL subgroup, where serine/threonine kinase 11 (*STK11*), Kelch-like ECH-associated protein 1 (*KEAP1*) or liver kinase B1 (*LKB1*) is identified, is usually associated with low percentages of PD-L1; and the KC subgroup, which is characterized by inactivation of CDK2A/B, is associated with a mucinous histology [60, 61]. In contrast, it is very rare to find *EGFR* mutations, thus *KRAS* and *EGFR* mutations are considered mutually exclusive.

The used technique to identify *KRAS* mutations is usually PCR. Thus far, their detection in isolation is not recommended, but should be included in NGS panels [62].

After years without effective therapies, several inhibitors have been developed that have shown activity in phase II trials against the *KRAS*^G12C^ mutation, such as sotorasib and adagrasib [63, 64]; therefore, they have been approved by the FDA, although the benefit of these agents in monotherapy or in combination, and to which patients they should be administered, are being studied in phase III trials.

### MET

Oncogenic activation of the mesenchymal epithelial transition factor (*MET*) gene in NSCLC can occur mainly by amplification (1–5%) or by the presence of mutations in exon 14 (3–4%) that reduce degradation of the *MET* protein [9]. In NSCLC with a sarcomatoid morphology, the frequency of mutations in exon 14 can be up to 22% [65]. Between 5 and 20% of patients with *EGFR* mutations acquire resistance to *EGFR*-TKIs through *MET* amplification [9].

Currently, the results of several clinical trials demonstrate the activity and tolerability of oral drugs such as capmatinib, tepotinib and savolitinib in patients with mutations in exon 14, and capmatinib and tepotinib have already been approved by the EMA [9]. Conjugated antibodies such as telisotuzumab vedotin, bispecific antibodies such as amivantamab and others are also being studied in patients with *MET* amplification.

The technique of choice to study *MET* amplification is FISH, since it allows an estimation of the increase in the number of copies and clonal amplification with more precision. Due to the heterogeneity of mutations in exon 14, the optimal detection method in this case is NGS. An NGS panel with sufficient coverage should be used. To avoid false-negative results, an RNA panel is recommended [9]. *MET* overexpression by amplification or mutation of the gene can be detected, but the predictive value of MET IHC is still controversial [7, 9].

#### HER2

Overexpression, amplification and mutations of *HER2* can be found in NSCLC, which are identified in 3–38%, 3% and 1–4% of patients, respectively [66]. The most frequent mutations are insertions in exon 20 (the tyrosine kinase domain), with the insertion/duplication of the four amino acids tyrosine, valine, methionine and alanine (YVMA) at codon 776 (YVMA 776–779 ins) being the most frequent (80–90%) [66, 67]. These mutations are mainly associated with patients with adenocarcinoma, nonsmoking patients and women [68]. The most recent data suggest that these mutations are the best predictors of a clinical benefit with anti-*HER2* therapies (e.g., trastuzumab deruxtecan), regardless of the type of mutation and the presence of overexpression and amplification [67].

Regarding the methodologies for evaluating *HER2* status, DNA- or RNA-based NGS is the most appropriate method to select patients compared to IHC and FISH [67]. Amplification has been described as a resistance mechanism following targeted treatments [66].

## Immune biomarkers with potential value

The tumour mutation burden (TMB) refers to the number of somatic mutations present in the tumour, excluding polymorphisms and germline mutations from all variants, expressed per megabase in the studied exome. The mutations acquired by tumour cells may lead to abnormal protein structure, and consequently, to the expression of neoantigens that can elicit an immunotherapy response. Interestingly, there is no clear correlation between the expression of PD-L1 and TMB [69]. Many studies have shown that high TMB in tumours results in a better therapeutic effect with anti-PD-1/PD-L1 immunotherapy, including in some lung cancers [69], but there is no definitive validation for the use of this immunotherapy in clinical practice. However, exploratory analysis of the Keynote 042 trial suggests that among patients with tumours expressing PD-L1 in ≥ 50% of cells, only those whose TMB was higher than the median exhibited any therapeutic benefit with PD-1/PD-L1 inhibitors as compared to chemotherapy [70]. In fact, TMB is not a reliable predictor of outcome for NSCLC or SCLC treated with chemotherapy plus ICI blockade or dual immuno-oncology regimens.

With regard to testing for TMB, targeted NGS is considered to be a good alternative to more complex massive sequencing, and some recent data have validated the use of large panels [71, 72]. Harmonization studies are still required to validate the interconnectivity between different NGS studies, the heterogeneity of the numbers of included genes, horizontal coverage, the required optimal depth, the chemical sequencing type and the bioinformatic algorithms used [71]. If eventually drugs are approved based on TMB cut-offs, the harmonization efforts underway could be very useful. Detection of TMB in the blood (bTMB) is feasible, but robust data on its clinical utility are still lacking.

Microsatellite instability-high/deficient MisMatch repair (MSI-H/dMMR) predicts the efficacy of ICIs in gastric cancer and colon cancer. However the incidence of MSI-H/dMMR in lung cancer is low [73], and further investigation is needed to determine whether MSI-H/dMMR can be used as a predictive biomarker in this context. At present, the standard measure commonly used to judge MSI-H is the Bethesda method [74]. Of note, patients with MSI-H have a higher probability of having high TMB, but not vice versa [75].

The predictive role of genomic aberrations underlying lung cancer have also been investigated [76]. Gene alterations typically perceived as associated with immunotherapy response include those in *TP53* or *KRAS*, and on the contrary aberrations affecting *EGFR*, *ALK*, *ROS*, *RET*, *KEAP1* or *LKB1* are less likely to be associated to checkpoint blockade benefit [61, 76]. In any case, at present, the available data do not support treatment recommendations based only on those genomic determinations.

Tumour inflammatory biomarkers such as related gene signatures or tissue cell content (T cell subtypes, myeloid cells, etc.) are investigational at present as they are peripheral blood-related immunotherapy and microbiome efficacy biomarkers.

## Prioritizing the use of biological samples to achieve an accurate diagnosis

**Fig. 1:**
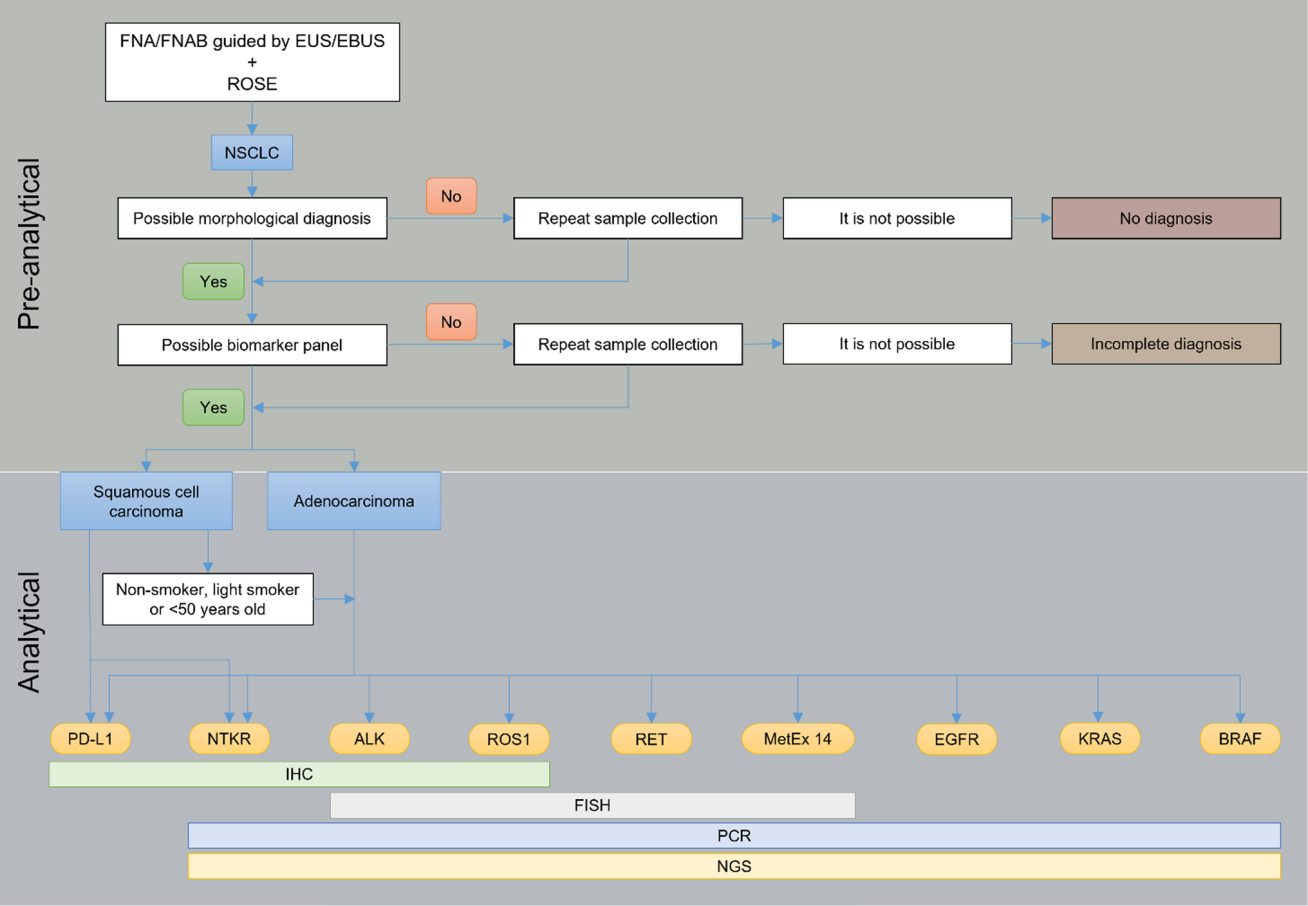
Update of the small sample diagnostic algorithm in patients with NSCLC. *ALK* anaplastic lymphoma kinase, *BRAF* B-Raf proto-oncogene, *EBUS* endobronchial ultrasound, *EUS* endoscopic ultrasound, *EGFR* epidermal growth factor receptor, *FISH* fluorescence in situ hybridisation, *FNA* fine needle aspiration, *FNAB* fine needle aspiration biopsy, *IHC* immunohistochemistry, *KRAS* kirsten rat sarcoma virus, *MetEx 14* mesenchymal epithelial transition factor exon 14, *NGS* next-generation sequencing, *NSCLC* non-small cell lung cancer, *NTRK* neurotrophic tyrosine receptor kinase, *PCR* polymerase chain reaction, *PD-L1* programmed death ligand-1, *ROS1* c-ros oncogene 1, *RET* rearranged during transfection, *ROSE* rapid on site evaluation

A high percentage of patients with NSCLC are diagnosed in advanced stages. These patients are not eligible for surgery; thus, the diagnosis is established through small biopsies and cytological samples. The development of imaging techniques that guide fine needle aspiration (FNA) and fine needle aspiration biopsy (FNAB) allows the acquisition of high-quality samples in the quantities necessary to perform a complete diagnosis, both morphologically and via biomarkers (Fig. 1) [5].

International guidelines recommend that regardless of the type of sample [77]: (1) a precise morphological diagnosis should be determined (i.e., NSCLC subtype); (2) the diagnosis of NSCLC not otherwise specified (NSCLC-NOS) should account for less than 10% of diagnoses with small samples/cytology; (3) IHC/immunocytochemistry (ICC) should be used judiciously; and iv) samples should be saved for biomarker studies.

In the case of FNA/FNAB guided by imaging techniques, the use of rapid on-site evaluation (ROSE) is recommended. In addition to assessing whether a sample is sufficiently adequate to facilitate a diagnostic approach, ROSE also allows control of the entire pre-analytical phase and in situ preparation of the sample for analysis of the necessary biomarkers according to the preliminary diagnostic impression [5, 78].

In the case of cytological samples, samples should be handled in situ for proper processing by the following: (1) smears with immediate 96% alcohol fixation; (2) air-dried smears stained with Giemsa^®^/Diff-Quik^®^; (3) cell blocking; and (4) washing a needle in liquid cytology fixative (which provides good RNA conservation).

All these types of cytological samples are useful for biomarker detection by IHC/ICC, FISH and PCR-based techniques [5, 79–81].

IHC/ICC offers excellent results, which are comparable to those obtained by cell block biopsy and in smears previously stained with Papanicolaou. Unstained cytological smears stained with Giemsa^®^/Diff-Quik^®^ and Papanicolaou are excellent substrates for FISH. Whole nuclei are analysed and, therefore, the signals observed are real, with no truncation effect due to the cutting of the paraffin samples. Moreover, DNA and RNA are of better quality in samples not fixed in formalin.

Molecular studies are usually less challenging on surgical specimens due to a greater amount of tissue. However, difficulty remains, and surgical pieces should be adequately fixed within 24–48 h. Necrotic areas should be avoided, and detection should be performed on samples with at least 30% viable tumour cellularity. In addition, the pieces should be adequately cut according to macroscopic protocols (e.g., white paper on pathology), including sufficient number of sections of the tumour. Tumours no larger than 3 cm should be included in their entirety. A good histological study is the first biomarker, since it will determine the histological subtype and guide further molecular detection (superimposable to that indicated above for small biopsy and cytology). Some histological subtypes are associated with different molecular alterations, although any clinical or histological variable should not be considered exclusive for biomarker detection in lung cancer. Another debate concerns molecular detection in tumours with different histological subtypes (a very common occurrence in adenocarcinomas). In these cases, testing samples corresponding to the most frequent subtype would be convenient, and detection in secondary subtypes can be added, especially if they present mucinous differentiation, in clear cells or signet-ring cells or in the presence of psammoma bodies.

**Fig. 2:**
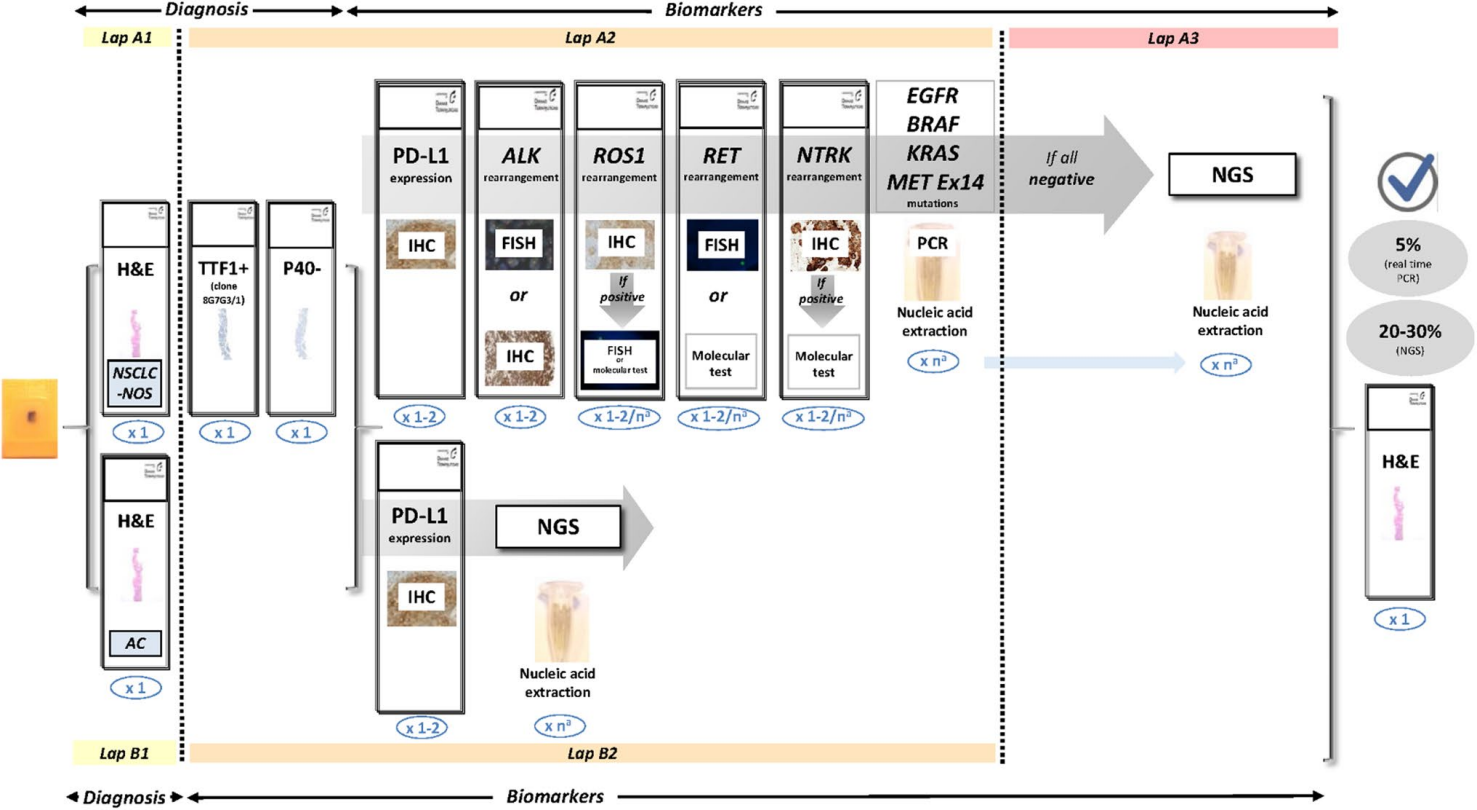
Updated protocol for multiple biomarker testing on samples from patients with NSCLC. The number of sections for each test is shown in blue. ^a^The requirements for nucleic acid extraction for individual molecular testing or for extended genetic panels (NGS) are variable. Figure modified from Conde et al. (confidential, submitted). *AC* adenocarcinoma, *ALK* anaplastic lymphoma kinase, *EGFR* epidermal growth factor receptor, *FISH* fluorescence in situ hybridisation, *H&E* haematoxylin and eosin, *IHC* immunohistochemistry, *KRAS* kirsten rat sarcoma virus, *MET* mesenchymal epithelial transition factor, *NGS* next-generation sequencing, *NTRK* neurotrophic tyrosine receptor kinase, *NSCLC-NOS* non-small-cell lung carcinoma—not otherwise specified, *PCR* polymerase chain reaction, *RET* rearranged during transfection, *ROS1* c-ros oncogene 1, *PD-L1* programmed death ligand-1

For all the biomarker analysis methods cited above, Fig. 2 shows an update of the protocol to be followed to analyse a biological sample of NSCLC.

## The role of NGS in NSCLC

In recent years, the need for multigenic tests in patients with lung cancer has increased, including alterations in oncogenic factors, computations or resistance mechanisms [82]. NGS allows the sequencing of long and complex genes and multiple genes in a patient sample to identify alterations in factors and targets, minimizing the use of tissues in a short period and their use in daily clinical practice.

The European Society for Medical Oncology (ESMO) has established recommendations on whether multigenic tumour NGS can be used and how to profile metastatic cancers following the classification of the Scale for Clinical Actionability of Molecular Targets (ESCAT) [83]. ESCAT is a framework that classifies the correspondence between a drug and genomic alterations according to their ability to act at the following three levels [84]: (1) from the perspective of public health; (2) from the perspective of academic clinical research centres; and (3) at the level of each individual patient.

With regard to lung cancer, the general recommendations for daily practice consider that a tumour or plasma sample from a patient with advanced nonsquamous NSCLC is profiled using NGS technology to detect level I ESCAT alterations (the alteration-drug pairing is associated with a better outcome in clinical trials, ready for routine use). The tests should include *EGFR*, *ALK*, *ROS1*, *BRAF*, *RET*, *HER2*, *NTRK*, *KRAS* and *MET*. In addition, for clinical research centres, multigene sequencing is strongly recommended for innovative drugs and clinical trials, as opposed to use in individual patients, for whom few clinically significant findings are expected with NGS [83].

Selection of the size of the NGS panel depends on the type of alterations to be studied, the response time required and the costs that can be expected [85, 86]. Each service should implement the panel that best meets its needs and be very familiar with its coverage to be able to expand molecular studies if the initial results are completely negative. Thus, to identify treatable fusions, the use of RNA panels is recommended because they are more sensitive than those that exclusively use DNA [87].

The starting nucleic acids are obtained mainly from formalin-fixed and paraffin-embedded samples (including both tissue samples and cell blocks from cytological samples) or from the nucleic acids present in the plasma. In the first stage of sample preparation, an exhaustive review of all the material from each patient is essential for the selection of the most appropriate paraffin block considering the pre-analytical variables (insufficient fixation and all fixatives that are not neutral buffered formalin at 10% must be avoided) and the percentage of tumour cellularity (optimal cutoff point: equal to or greater than 30%) [88].

The two methodological approaches of NGS most implemented in clinical practice are bridge amplification (Illumina^®^, San Diego, CA, USA) and emulsion PCR (ThermoFisher Scientific^®^, Waltham, MA, USA), which each have strengths and weaknesses. Among the advantages of Illumina^®^ bridge amplification, this method allows identification of unknown alterations and the use of larger gene panels, while ThermoFisher Scientific^®^ emulsion PCR requires less starting material and yields molecular results with shorter response times [54, 89].

The molecular findings obtained should be reflected in the NGS results report, together with the relevant conclusions regarding the tumour of each patient. The results should be discussed in a multidisciplinary committee, since increasing evidence indicates that this practice improves clinical outcomes [90].

## The role of liquid biopsy in NSCLC

The concept of LB encompasses tests performed on a sample of peripheral blood or other biological fluid with the objective of detecting circulating tumour cells (CTCs) or fragments of nucleic acids from a tumour, such as circulating free DNA (cfDNA), ctDNA, circulating exosomes, platelet RNA and circulating tumour RNA (ctRNA), which can be isolated from blood (plasma) or urine, pleural fluids, ascites, cerebrospinal fluid (CSF) and saliva. LB has high specificity (96%), but its sensitivity is only 66% [91]. Therefore, a negative result is not definitive and requires tissue confirmation.

Therefore, its role is complementary to biopsy, and current recommendations are based on two clinical contexts when a tissue sample is limited or insufficient: (a) detection of molecular sensitive alterations and (b) detection of resistance mechanisms after progression on a TKI [21, 92]. A third context would be treatment efficacy monitoring based on the ctDNA load for minimal residual disease (MRD), an attractive approach that is not yet well established technically (quantification units need to be established), but where fluctuating levels of circulating nontumour DNA can affect the results [92]. Its use in early diagnosis presents difficulties due to low sensitivity in localized disease. Essentially, the greatest development has been in *EGFR* mutations, but currently, LB is being incorporated into tests for other molecular alterations, although the detection of gene rearrangements from circulating RNA continues to be a technical challenge awaiting resolution [21].

Some technical requirements of LB are the need for larger than usual blood volumes (two 10-ml tubes). Plasma is preferred rather than serum for nucleic acid extraction. The maximum waiting time until plasma extraction is 2 h for tubes with ethylenediaminetetraacetic acid (EDTA) and 3 days for tubes with special preservatives (Streck^®^). Blood should not be frozen before plasma extraction. DNA extraction should be performed with protocols designed for small and fragmented DNA [92]. Notably, up to 10% of people over 65 years of age present clonal haematopoiesis phenomena that may be misinterpreted as false-positive findings [93].

The use of techniques with high sensitivity, such as digital PCR, is recommended. In the case of NGS, good agreement is evident for tissue results, except for the variants that are found with an allelic frequency lower than 1% [94]. However, tools such as the unique molecular identifier (UMI) can be used to optimize detection. Two commercial NGS platforms (Guardant360^®^ and FoundationOne Liquid CDx) have FDA approval for the analysis of solid tumours, including lung carcinomas [21].

In short, LB will be progressively incorporated into molecular diagnosis, treatment monitoring, MRD detection and early diagnosis as soon as prospective studies confirm its clinical utility.

## Main requirements for implementing good quality control

Molecular testing is becoming an essential diagnostic tool and a part of standard management in cancer patients. Both laboratories and pathologists face new challenges in order to meet this novel requirement in patient care. Pathology laboratories must incorporate reliable methods to ensure optimal sample quality and processing to reduce the risk of errors in molecular biology tests [95]. Furthermore, practicing pathologists need to go beyond diagnosis and classification in order to produce information that will be required to guide treatment accurately and to do so in a timely manner [96].

The results of predictive biomarkers often determine which therapy (e.g., chemotherapy, immunotherapy, or targeted therapy) patients receive. Laboratory errors may therefore result in wrong or suboptimal treatment decisions and consequently harm the patient. To assure high-quality testing, laboratories must have a quality assurance system in place and comply with relevant international standards from certified organizations such as the International Organization for Standardization (ISO), the College of American Pathologists (CAP), or the Clinical Laboratory Improvement Amendments (CLIA) (Table 3) [96–100].

**Table 3:** Examples of European quality assurance schemes

Supplier	Name	Starting material	Aim	Format
EMQN	Molecular testing of cfDNA in plasma for *EGFR* gene mutations (pilot)	Plasma containing cfDNA	Mutations in the *EGFR* gene	5 mock clinical cases with matching samples
	Molecular testing in lung cancer	Mix of real tissue and artificial FFPE materials	Mutations in the *EGFR*, *PIK3CA*, *KRAS* and *BRAF* genes	10 mock clinical cases with matching samples
	DNA Sequencing – NGS (vSomatic)	DNA sample derived from FFPE material	Any NGS strategy can be used	1 mock clinical case with matching samples
	Oncogene panel testing	Rolled sections of FFPE materials	Mutations in the *EGFR*, *PIK3CA*, *KRAS*, *HRAS*, *NRAS*, *KIT*, *TP53* and *BRAF* genes	3 mock clinical cases with matching samples
ESP	*ALK* FISH	Slides	*ALK* rearrangements	5 resections, 5 digital cases
	ALK IHC	Slides	*ALK* rearrangements	5 resections
	*EGFR*, *KRAS* (optional), *BRAF* (optional)	Slides/rolled sections	Mutations	10 resection specimens, possible cell-line
	*ROS1* FISH	Slides	*ROS1* rearrangements	5 resections or possibly cell-lines, 5 digital cases
	ROS1 IHC	Slides	*ROS1* rearrangements	5 resections or possibly cell-lines
	PD-L1	Slides	PD-L1 overexpression	8 resections (TMAs) and 4 digital cases
	*MET* EQA scheme (ex 14 skipping) for DNA and RNA	Slides/rolled sections	*MET* exon 14 mutations	5 resections
NordiQC	Companion PD-L1	Slides	PD-L1 overexpression	1 preparation with multiple cases + 1 *in-house* case
SEAP	ALKanza MODULE	Slides	*ALK* rearrangements	1 slide with 4 cases + 1 *in house*
	*EGFR*	Slides/rolled sections	*EGFR* mutations	4 consecutive slides
UKNQEQAS	NSCLC ALK IHC	Slides	*ALK* and *ROS1* rearrangements	1 slide with several cases + 1 *in house*
	NSCLC *ALK/ROS1* FISH (pilot)	Slides	*ALK* and *ROS1* rearrangements	1 slide with several cases + 1 *in house*
	NSCLC PD-L1 IHC (pilot)	Slides	PD-L1 overexpression	1 slide with several cases + 1 *in house*
Gen QA	Lung cancer	Slides/rolled sections	*EGFR, ALK* (optional), *KRAS* (optional), *BRAF* (optional)	5–4 cases
	ctDNA (pilot)	Plasma	*EGFR* mutations	5 cases
	Additional lung cancer biomarkers	Slides/rolled sections	*ROS1*, *RET* and *MET* (amplification)	4 cases

*ALK* anaplastic lymphoma kinase, *BRAF* B-Raf proto-oncogene, *cfDNA* circulating free DNA, *ctDNA* circulating tumour DNA, *EGFR* epidermal growth factor receptor, *FFPE* formalin-fixed paraffin-embedded, *FISH* fluorescence in situ hybridisation, *HRAS* Harvey Rat sarcoma virus, *IHC* immunohistochemistry, *KIT* proto-oncogene receptor tyrosine kinase, *KRAS* kirsten rat sarcoma virus, *MET* mesenchymal epithelial transition factor, *NGS* next-generation sequencing, *NRAS* neuroblastoma ras viral oncogene homolog, *NSCLC* non-small cell lung cancer, *PD-L1* programmed death ligand-1, *PIK3CA* phosphatidylinositol 4,5-bisphosphate 3-kinase catalytic subunit alpha, *RET* rearranged during transfection, *ROS1* c-ros oncogene 1, *TMAs* tissue microarrays, *TP53* tumour protein 53

Progress in personalized medicine is limited, in part, because of the lack of standardized European and international documentation and insufficient guidelines for pre-analytical workflows. The pre-analytical process has recently been examined in some detail by the SPIDIA^®^ project (http://www.spidia.eu). The following data were considered necessary to issue a report in accordance with good practice guidelines (Table 4) [96]: (1) patient identification: the patient must be identified correctly—laboratories require a minimum of two unique patient identifiers plus a unique sample identifier on the request form and the report; (2) reporting style and content: long reports are rarely read in full, and length matters; one page or, better still, single-screen reports are preferred, provided that they are legible. Clear presentation of the results, the test(s) performed, and any limitations of the tests (e.g., whether all possible mutations or a selection of the more common ones were tested) must be included; (3) interpretation: the result of the test must be described and be provided with an appropriate interpretation, particularly when this involves a treatment decision; (4) integrated reporting: the need for integrating patients’ results is widely acknowledged. As gene panel testing becomes more widespread, the results of different gene tests should be merged into a single report. Also, results from several pathology specialties on individual patients need to be integrated into the same report.

**Table 4:** Proposed pathology results report content

Identification of the patient and the doctor who ordered the test (or, failing that, the authorising person)
Pathological diagnosis
Type of specimen submitted Previous treatment (yes/no) Timing of biopsy (initial/relapse/progression) Date on which the specimen was collected
The external code in the case of referral centres
The medium in which the specimen was received (fresh, frozen, paraffin-embedded, etc.)
The anatomical origin of the specimen
The order date, the specimen receipt date and the date on which the results were issued
The biomarker test method used, specifying detectable mutations and/or other abnormalities. In the case of commercial kits, the commercial name, the batch number and whether they are an approved ‘in vitro diagnostics’ product should be stated
Name of the platform used and expiration date of the product The quality of the sample, specifying the percentage of cancer cells and whether the sample was enriched by micro- or macrodissection, as well as DNA concentration and purity
Comments about the adequate or inadequate nature of the sample
The test result, defining the type of molecular abnormality detected or the absence of molecular abnormalities
Identification of the professional responsible for the test (all phases)
Identification of the laboratory supervisor (optional)
Any additional information or comments of interest to the doctor who ordered the test
Accreditation or participation in quality programs

Surgical Pathology services should obtain accreditation on quality assurance. We believe that all laboratories providing molecular pathology services should have laboratory accreditation according to ISO 15189 or their national equivalent. Accreditation provides patients, staff, service users and commissioners with evidence of laboratory competence.

## Conclusions

Currently, in patients with NSCLC, a clear genomic diagnostic strategy that allows the establishment of optimal therapeutic indications for each must be defined.

With this objective, in the new consensus of the SEOM and the SEAP, the following recommendations are proposed: (1) *EGFR*, *BRAF*, *KRAS* and *MET* mutations, *ALK*, *ROS1*, *RET* and *NTRK* translocations and PD-L1 expression must be detected in NSCLC; (2) other emerging biomarkers such as the *HER2* mutation and immune biomarkers such as TMB, MSI, *STK11* and *KEAP1* are recommended, especially if NGS is available; (3) molecular detection can be performed at any stage of NSCLC or in clinically selected patients, and new therapeutic indications that require this information can be established; (4) the availability of NGS strongly facilitates molecular diagnosis in a precise and effective manner, and its use should be immediately generalized; (5) LB has an increasing role in molecular diagnosis, especially if tissue is limited, and its role in the follow-up of treatment is also promising, both in MRD detection and early diagnosis; (6) a tumour sample must be appropriately analysed for correct prioritization of the molecular detection test to be performed, and good quality control throughout the process is essential; (7) adequate multidisciplinary collaboration between the different professionals involved is needed to achieve the highest quality in the diagnostic process and in the detection of the best therapeutic approach for each patient with NSCLC at any stage of the disease.

## Data Availability

Not applicable.
