# Correction to: New update to the guidelines on testing predictive biomarkers in non‑small‑cell lung cancer: a National Consensus of the Spanish Society of Pathology and the Spanish Society of Medical Oncology

**DOI:** 10.1007/s12094-023-03103-x

**Published:** 2023-02-08

**Authors:** Dolores Isla, Maria D. Lozano, Luis Paz-Ares, Clara Salas, Javier de Castro, Esther Conde, Enriqueta Felip, Javier Gómez-Román, Pilar Garrido, Ana Belén Enguita

**Affiliations:** 1 Lozano Blesa University Clinical Hospital, IIS Aragón, Spanish Society of Medical Oncology (SEOM), C. de San Juan Bosco, 15, 50009 Zaragoza, Spain; 2 Clínica Universidad de Navarra, Spanish Society of Cytology (SEC), Spanish Society of Pathology (SEAP), Pamplona, Spain; 3 12 de Octubre University Hospital, Spanish Society of Medical Oncology (SEOM), Madrid, Spain; 4 Puerta de Hierro University Hospital, Spanish Society of Pathology (SEAP), Madrid, Spain; 5 La Paz University Hospital, Hospital La Paz Institute for Health Research (IdiPAZ), Spanish Society of Medical Oncology (SEOM), Madrid, Spain; 6 12 de Octubre University Hospital, Research Institute 12 de Octubre University Hospital (I+12), Spanish Society of Pathology (SEAP), Madrid, Spain; 7 Vall d’Hebron University Hospital, Spanish Society of Medical Oncology (SEOM), Barcelona, Spain; 8 Cantabria University, Marqués de Valdecilla University Hospital, Valdecilla Biomedical Research Institute (IDIVAL), Spanish Society of Pathology (SEAP), Santander, Spain; 9 Ramón y Cajal University Hospital, Spanish Society of Medical Oncology (SEOM), Madrid, Spain; 10 12 de Octubre University Hospital, Spanish Society of Pathology (SEAP), Madrid, Spain

**Correction to: Clinical and Translational Oncology** 10.1007/s12094-022-03046-9

In this article the caption to Fig. 2 was incorrect and should have been as follows.

**Fig. 2** Updated protocol for multiple biomarker testing on samples from patients with NSCLC. The number of sections for each test is shown in blue. aThe requirements for nucleic acid extraction for individual molecular testing or for extended genetic panels (NGS) are variable. Figure modified from Conde et al. (confidential, submitted). *AC* adenocarcinoma, *ALK* anaplastic lymphoma kinase, *EGFR* epidermal growth factor receptor, *FISH* fluorescence in situ hybridisation, *H&E* haematoxylin and eosin, *IHC* immunohistochemistry, *KRAS* kirsten rat sarcoma virus, *MET* mesenchymal epithelial transition factor, *NGS* next-generation sequencing, *NTRK* neurotrophic tyrosine receptor kinase, *NSCLCNOS* non-small-cell lung carcinoma—not otherwise specified, *PCR* polymerase chain reaction, *RET* rearranged during transfection, *ROS1* c-ros oncogene 1, *PD-L1* programmed death ligand-1

The original article has been corrected.

